# Acceptance of the Disease in Patients Diagnosed with Neovascular Age-Related Macular Degeneration Depending on Visual Parameters—Before and After a Series of Seven Intravitreal Injections

**DOI:** 10.3390/jcm14020447

**Published:** 2025-01-12

**Authors:** Marta Nowak, Anna Maria Cybulska, Daria Schneider-Matyka, Elżbieta Grochans, Ireneusz Walaszek, Mariusz Panczyk, Grzegorz Józef Nowicki, Kamila Rachubińska

**Affiliations:** 1 Department of Nursing, Faculty of Health Sciences, Pomeranian Medical University in Szczecin, 71-210 Szczecin, Poland; marta.nowak@pum.edu.pl (M.N.); daria.schneider.matyka@pum.edu.pl (D.S.-M.); elzbieta.grochans@pum.edu.pl (E.G.); ireneusz.walaszek@pum.edu.pl (I.W.); kamila.rachubinska@pum.edu.pl (K.R.); 2 Department of Education and Research in Health Sciences, Faculty of Health Sciences, Medical University of Warsaw, 02-091 Warsaw, Poland; mariusz.panczyk@wum.edu.pl; 3 Department of Family and Geriatric Nursing, Faculty of Health Sciences, Medical University of Lublin, 20-059 Lublin, Poland; gnowicki84@gmail.com

**Keywords:** age-related macular degeneration, anti-VEGF, treatment, acceptance of illness

## Abstract

**Background:** Age-related macular degeneration (AMD) is a progressive, chronic eye disease with no permanent cure currently available. Symptoms of the disease, including distorted and blurred vision and gradual loss of central vision, significantly aggravate patients’ daily functioning. The purpose of this study was to assess the acceptance of the disease among patients diagnosed with neovascular age-related macular degeneration before treatment and after receiving seven intravitreal injections and to determine how it was related to the values of visual parameters. **Methods:** This survey-based study was carried out using the author’s questionnaire and a standardized research tool, the Acceptance of Illness Scale (AIS). It also involved the analysis of the patients’ medical records. **Results:** The study included 121 patients (121 eyes), including 60 women and 61 men. The age range of the participants was 51–90 years. The mean and median age of the participants was 75 years. After undergoing a series of intravitreal injections, statistically significant improvements were observed in the degree of illness acceptance according to the AIS score. Data analysis revealed that the degree of disease acceptance was significantly related to visual acuity and contrast sensitivity. **Conclusions:** The acceptance of the disease among the study participants from the beginning of the therapy until receiving a series of seven intravitreal injections was at an average level. Acceptance of the disease was better before the beginning of the therapy, due to higher values of corrected visual acuity, and after the therapy, because of higher values of contrast sensitivity and corrected visual acuity.

## 1. Introduction

Age-related macular degeneration (AMD) develops in people over the age of 50 years, and its risk increases with age [1]. Clinical classification of AMD defines three stages according to the severity of fundus lesions: early, intermediate, and late AMD. Late AMD can be either neovascular AMD or atrophic AMD [2].

Degenerative changes in the retinal photoreceptors, Bruch’s membrane, and choriocapillaris underlie AMD pathogenesis. The aforementioned structures are responsible for high-resolution vision, color vision, peripheral vision, and twilight vision, and the pathologies arising within them are reflected in the symptoms experienced by the patients. The main visual abnormalities found in AMD patients are metamorphopsia, that is, seeing objects as distorted, warped, or wavy. What is responsible for its development is caused by a combination of changes in the processing of visual stimuli in the cerebral cortex and structural changes in the retina (including displacement of the photoreceptor layer caused by the accumulation of fluid from abnormal vessels) [3]. Visual disturbances in AMD patients depend on the severity of the disease. In addition to metamorphopsia, visual acuity is also decreased, alongside central scotoma—the perception of an oval or irregular dark spot in the field of vision; macropsia—the perception of an object as larger than it actually is; micropsia—the perception of an object as smaller than it actually is; and dyschromatopsia—abnormal color perception that may manifest itself in the overall vision of colors with lower saturation than in reality [4,5].

Currently, there is no therapy that causally and completely eliminates the disease and its source. Monitoring of the non-exudative form of AMD, commonly referred to as the dry form, involves regular self-observation of the patient and systematic ophthalmological check-ups for the early detection of potential macular neovascularization. Treatment of the dry form, which can progress to the exudative (or wet) form, is reduced to non-pharmacological therapy, namely supplementation and lifestyle modification. In the case of the active neovascular form, pharmacotherapy is a crucial addition to non-pharmacological methods. It primarily includes anti-vascular endothelial growth factor (anti-VEGF) therapy, which is the gold standard for the treatment of exudative AMD. Treatment involves intravitreal administration of agents that reduce intraocular concentrations of VEGF, a proangiogenic factor that plays a key role in the formation of pathological neovascularization in the course of exudative AMD [6,7,8,9,10,11].

Acceptance of the disease plays an important role in the context of AMD as it affects the psychological adaptation of patients. People who accept their illness tend to have a higher quality of life because of less stress, anxiety, and depression associated with chronic conditions [12,13]. Acceptance of AMD may also lead to greater motivation to follow medical recommendations, which improves visual parameters such as visual acuity, contrast sensitivity, and the presence and severity of metamorphopsia and central scotoma, which in turn translates into better functioning and comfort of life. Adaptation to vision loss is easier for those who accept their disease, as they are more likely to seek support and learn new skills such as using vision aids. Acceptance of AMD also results in greater involvement in social and professional activities, which are important for maintaining high self-esteem and independence [14,15]. Psychological support, education, and involvement in the treatment process are key to improving the lives of AMD patients [16]. The purpose of this study was to assess the acceptance of the disease among patients diagnosed with neovascular age-related macular degeneration before treatment and after receiving seven intravitreal injections and to determine how it was related to the values of visual acuity and contrast sensitivity, as well as the presence and severity of metamorphopsia and central scotoma.

## 2. Materials and Methods

### 2.1. Settings and Design

The study was conducted at University Clinical Hospital No. 2 of the Pomeranian Medical University in Szczecin among patients diagnosed with exudative AMD (H35.3) who qualified for treatment under the drug program reimbursed by the National Health Fund in Poland. All patients signed the written informed consent form and agreed to participate in scientific research. The study included 132 patients, of whom 6 died, 1 withdrew from treatment, and 4 were excluded from the drug program due to failure to meet the criteria for therapy. Finally, 121 patients (121 eyes) were analyzed. The study was approved by the Bioethics Committee of Pomeranian Medical University in Szczecin (KB-006/02/2022/Z, approval date: 21 January 2022) and was conducted in accordance with the Declaration of Helsinki.

The inclusion criteria were age-related macular degeneration (neovascular form) diagnosed on the basis of the ICD-10 criteria (H35.3), participation in the National Health Fund drug program “Treatment of patients with retinal diseases”, giving informed consent to participate in the study, having no mental conditions that may cause depressive or anxiety disorders unrelated to AMD, and having no neurodegenerative conditions (such as Alzheimer’s disease or dementia) preventing the patient from completing the questionnaire independently and consciously.

The exclusion criteria for the study were as follows: lack of consent to participate in the study, lack of consent to undergo treatment under the drug program, diagnosis of mental disorders that could lead to depressive or anxiety symptoms unrelated to age-related macular degeneration (AMD), or the presence of neurodegenerative diseases, such as Alzheimer’s disease or dementia, that would hinder the patient’s ability to independently and consciously complete the questionnaire.

In Poland, patients with neovascular age-related macular degeneration (nAMD) have the option of treatment under the drug program “Treatment of patients with retinal diseases”, financed by the National Health Fund. However, inclusion in the drug program requires meeting a number of criteria, both during qualification and during the period of participation. The inclusion criteria are as follows:

1. Active (primary or secondary) subretinal neovascularization occupying more than 50% of the AMD lesion confirmed by optical coherence tomography (OCT) and optical coherence tomography angiography (a-OCT) or fluorescein angiography (AF).
2. Age over 45 years.
3. Total lesion size less than 12 disc areas.
4. Best corrected visual acuity in the treated eye of 0.2–0.8 determined according to the Snellen chart (or ETDRS equivalent, as appropriate).
5. Signed informed consent form.
6. No predominant geographic atrophy and hemorrhage.
7. No significant, permanent damage to foveal structure prior to treatment, defined as fibrosis, atrophy, or chronic discoid scar.

The exclusion criteria are as follows:

1. Hypersensitivity to the drug or any of the excipients.
2. Active ocular or periocular infection.
3. Active endophthalmitis
4. Pregnancy and lactation.
5. Adverse drug reactions that prevent its continued use.
6. Rhegmatogenous retinal detachment or macular hole grade 3 or 4.
7. Disease progression defined as follows:
  (a) Deterioration of visual acuity to <0.2 lasting more than two months;
  (b) Permanent damage to foveal structure preventing stabilization or functional improvement, defined as fibrosis, atrophy, or chronic discoid scar.

Failure to meet even one criterion at any stage entails the exclusion of the patient from the drug program and, thus, the inability to continue reimbursed treatment.

This research is part of a larger project entitled “The relationship between visual parameters and psychosocial aspects in patients diagnosed with neovascular age-related macular degeneration”.

The patients had eight visits, seven of which were qualified for intravitreal injections and one after completing the series of injections. The tests were performed at three visits under identical lighting conditions and using identical devices. The first check-up was carried out at the visit to qualify the patient for the first intravitreal injection (after approval from the Coordinating Team for the Treatment of Patients with Retinal Diseases) as part of the drug program. The second was at a visit qualifying the patient for the fourth intravitreal injection, after three intravitreal injections performed at 28-day intervals, in accordance with the treatment regimen under the drug program reimbursed by the National Health Fund. The third and final check-up was at a follow-up visit after seven intravitreal injections, performed at 56-day intervals, in accordance with the treatment regimen under the drug program reimbursed by the National Health Fund.

The examination performed during the check-up visits involved a visual acuity test without correction and with spectacle correction for distance vision; contrast sensitivity testing with spectacle correction for distance vision, OCT, and angio-OCT; and biomicroscopic examination of the anterior and posterior segments of the eye.

Each patient was surveyed and tested for the presence and severity of metamorphopsia and central scotoma using the Amsler grid (type 1 chart) with spectacle correction at the first and third (last) check-ups.

The risk of bias in the articles was assessed. Selection bias: To minimize the risk of selection bias, we applied clear and precise inclusion and exclusion criteria based on clinical parameters, such as the diagnosis of neovascular age-related macular degeneration (AMD). All patients were recruited from a single medical facility, which reduced potential differences arising from varying diagnostic or therapeutic standards. However, we acknowledge that the possibility of selection bias cannot be entirely excluded, and this limits the generalizability of our findings. Performance bias: To reduce performance bias, all patients were treated by the same medical team following a standardized protocol. This protocol was strictly adhered to during the administration of the seven intravitreal injections. We aimed to minimize differences in therapeutic approaches between patients, which could impact the results. However, potential differences in patient compliance with recommendations might have influenced the outcomes, which remains a limitation of the study. Detection bias: The risk of detection bias was minimized through the use of objective methods for measuring visual parameters (such as visual acuity and changes observed in OCT imaging). All measurements were conducted by trained specialists, and the diagnostic tools used were standardized. Attrition bias: Among the recruited patients, all completed the full treatment cycle and follow-up assessments, which minimizes the risk of attrition bias. Data from patients who might have withdrawn would have been analyzed following the “intention-to-treat” principle (if such cases had occurred). Reporting bias: The results are presented comprehensively and transparently, and all significant findings are included in the analysis and description. We also highlight the limitations of the study in the Discussion, which allows for a more thorough understanding of the results and their interpretation.

### 2.2. Research Instruments

This survey-based study was carried out using the author’s questionnaire and a standardized research tool, the Acceptance of Illness Scale (AIS) (described below). It also involved the analysis of the patients’ medical records.

- The author questionnaire regarded Sociodemographic data (sex, age, education, marital status, place of residence, employment status), use of stimulants (alcohol and nicotine), medical data (comorbidities, previous ophthalmic and laser procedures, ophthalmic diseases (cataract, glaucoma), and history of treatment with intravitreal injections.
- The Acceptance of Illness Scale (AIS) by Felton, Revenson, and Hinrichsen is used to estimate the level of acceptance of the disease. The tool consists of eight statements that describe the negative consequences of deteriorating health. Each statement is rated on a five-point Likert scale with ‘1’ denoting poor adaptation to the disease and ‘5’ its full acceptance. The final score is the sum of all points and ranges from 8 to 40, where a score of 8–18 points indicates low acceptance of the disease, a score of 19–29 points reflects a medium level of acceptance of the disease, and a score exceeding 29 points is equivalent to high acceptance of the disease. Cronbach’s alpha for the Polish version of the AIS was 0.82, reflecting its high reliability [17,18].

The following diagnostic tests were also performed during the course of the study:

- Testing distance visual acuity at a distance of five meters, under artificial lighting, without correction, and with spectacle correction, based on refraction measurement with an auto refractometer (Topcon KR-800), using illuminated Snellen optotypes (Topcon CC-100 series), whose values were converted to logMAR equivalents.
- Testing for the presence and severity of metamorphopsia and central scotoma using the Amsler grid (type 1 chart) at a distance of 30 cm from the face under artificial lighting, with spectacle correction selected based on refraction measurements using an auto refractometer (Topcon KR-800).
- Contrast sensitivity was tested using Pelli–Robson charts under artificial lighting at a distance of one meter, with spectacle correction for distance based on refraction measurement with an auto refractometer (Topcon KR-800).

### 2.3. Statistical Analysis

Descriptive statistics, encompassing means, standard deviations, medians, interquartile ranges, and coefficients of variation, were calculated for continuous variables. For categorical variables, frequencies and percentages were determined to provide a comprehensive overview of the sample characteristics.

According to the Polish Ophthalmological Society, the total population of patients with age-related macular degeneration (AMD) in Poland is relatively small [19] To account for this finite population, we employed the finite population correction when determining the minimum required sample size. Based on assumptions (a confidence level of 95% was applied, with a maximum margin of error of 7% and an estimated proportion of 0.5), we initially calculated a required sample size of approximately 195 participants to achieve the planned level of precision. Ultimately, 121 individuals met the full inclusion criteria and completed all study procedures, reflecting the practical constraints and recruitment challenges in this specific patient group. This reduction in sample size necessarily increased the margin of error from the intended 7% to roughly 8–9%. Nevertheless, the final sample remained adequate to analyze key clinical and psychosocial factors associated with AMD.

Comparative analyses of disease acceptance scores, assessed using the AIS, were conducted employing the paired *t*-test for dependent samples to evaluate differences between pre- and post-therapy measurements. For non-parametric comparisons of disease acceptance levels categorized as low, medium, or high, the Wilcoxon signed-rank test was utilized to accommodate the ordinal nature of these classifications.

To assess the associations between AIS scores and clinical predictors, multiple linear regression models were constructed. The predictors included corrected visual acuity, uncorrected visual acuity, and contrast sensitivity. The R2 adjusted value was reported to quantify the proportion of variance in AIS scores explained by the model’s predictors, thereby providing insight into the model’s explanatory power.

Associations between the presence and severity of visual disturbances, as evaluated through the Amsler test (specifically metamorphopsia, central scotoma, or both), and AIS scores were examined using multiple linear regression analysis. Regression coefficients (b), standard errors (SEs), standardized coefficients (β), and 95% confidence intervals (95% CIs) were calculated to interpret both the strength and direction of associations. For analyses involving categorical visual disturbances, a reference group comprising participants without visual disturbances was defined to facilitate meaningful comparisons.

The normality of continuous data distributions was verified using the Shapiro–Wilk test, while homoscedasticity was assessed using Levene’s test. Bonferroni correction was applied for multiple comparisons to mitigate the risk of Type I error inflation.

All statistical analyses were conducted using STATISTICA™ software version 13.3 (TIBCO Software, Palo Alto, CA, USA), adhering to a significance level of *p* < 0.05.

## 3. Results

### 3.1. Characteristics of the Study Sample

The study included 121 patients (60 women and 61 men) aged 51–90 years. The mean and median age of the subjects was 75 years. Of the participants, the largest group was people with vocational education (39.67%) and those with formal relationships (63.64%). People living in cities with more than 100,000 residents accounted for more than half of the respondents (58.68%). Retired individuals constituted the majority of the study group (86.78%). A total of 121 eyes were examined: 65 right (46.28%) and 56 left (53.72%). Among the study participants, 18 respondents (14.88%) admitted to using nicotine products, while the remaining 103 subjects (85.12%) denied being smokers. The most common comorbidities were hypertension (32.23%) and hypertension and diabetes comorbidity (26.45%). Only 17 (14.05%) of the 121 respondents were not diagnosed with any disease (Table 1 and Table 2).

### 3.2. Acceptance of the Disease as Measured by the AIS

The mean AIS scores were 22.12 ± 7.85 before therapy and 25.31 ± 7.83 after receiving seven intravitreal injections, with a maximum value of 40 points (Table 3).

There was a significant difference in the level of acceptance of the disease before and after therapy. The mean AIS score at the second measurement was significantly higher than the mean score on this scale at the first measurement (Δ = −3.20). This means that acceptance of the disease, as assessed by the AIS, was significantly higher (*p* < 0.001) at the eighth visit than before the start of therapy.

Before therapy, the largest group consisted of people with a medium level of acceptance of the disease (43.80%), followed by subjects with low (35.54%) and high (20.66%) levels of acceptance of the disease. At the eighth visit, the most numerous respondents had a medium level of disease acceptance (42.15%), followed by subjects with a high level of disease acceptance (33.06%) and patients with a low level of disease acceptance (24.79%). After receiving a series of seven intravitreal injections, acceptance of the disease as measured by AIS was significantly higher than that during the pre-therapy measurement (Table 4).

### 3.3. Acceptance of the Disease According to the AIS Depending on Visual Acuity and Contrast Sensitivity

Data analysis revealed that the degree of disease acceptance was significantly related to visual acuity and contrast sensitivity. Before therapy, a higher value of corrected visual acuity (a lower logMAR value) was associated with greater acceptance of the disease as measured by the AIS (*p* = 0.004). At the same time, no statistically significant relationships were observed between uncorrected visual acuity, contrast sensitivity, and acceptance of the disease according to the AIS before therapy (*p* = 0.265, *p* = 0.051). The three predictors analyzed together accounted for 19.6% of the variation in the level of acceptance of the disease according to the AIS (F(3, 117) = 10.8, *p* < 0.001) before therapy. Only one predictor, corrected visual acuity, had a significant negative effect (β = −0.30) on the AIS score, which means that higher visual acuity with correction was associated with a better quality of life according to the AIS score (Table 5).

Statistically significant relationships were also demonstrated after the subjects received seven intravitreal injections. Higher values of corrected visual acuity and contrast sensitivity were associated with a higher degree of disease acceptance according to the AIS score (*p* = 0.028, *p* = 0.009). However, there was no statistically significant relationship between uncorrected visual acuity and the degree of disease acceptance according to the AIS score. The three predictors analyzed together accounted for 21.8% of the variation in the level of acceptance of the disease according to the AIS (F(3, 117) = 12.1, *p* < 0.001) after the subjects received a series of seven intravitreal injections. Corrected visual acuity was the only predictor that had a significant negative effect (β = −0.29) on the AIS score. In contrast, the value of contrast sensitivity was the only predictor having a significant positive effect on the AIS score (β = 0.27), which means that higher values of corrected visual acuity and higher values of contrast sensitivity were associated with better acceptance of the disease according to the AIS (Table 6).

### 3.4. Acceptance of the Disease According to the AIS Depending on the Presence and Severity of Metamorphopsia and Central Scotoma (The Amsler Test)

Data analysis showed statistically significant relationships between the presence and severity of metamorphopsia and central scotoma on the Amsler grid and the degree of disease acceptance (Table 7 and Table 8). Before therapy, the analyzed predictor accounted for 6.03% of the variation in AIS score (F(3, 117) = 3.57, *p* = 0.016). Patients reporting the presence of central scotoma (β= −0.89) as well as both central scotoma and metamorphopsia (β = −0.85) were characterized by lower acceptance of the disease than their counterparts who reported no visual disturbances in the Amsler test. There were no statistically significant differences in the acceptance of the disease measured by AIS between patients reporting only metamorphopsia and those without any visual disturbances on the Amsler test (*p* = 0.100) (Table 7).

After seven intravitreal injections, the analyzed predictor accounted for 10.7% of the variation in AIS scores (F(3, 117) = 3.57; *p* < 0.001). Patients with metamorphopsia (β = −0.50), central scotoma (β = −1.03), and these two conditions (β = −1.25) had significantly lower acceptance of the disease than patients without any visual disturbances on the Amsler test (Table 8).

## 4. Discussion

Macular degeneration is a chronic, progressive, incurable disease. According to the World Health Organization (WHO), it is one of the leading causes of vision loss among elderly people, outclassing other serious ophthalmic diseases such as glaucoma or diabetic retinopathy. As the population ages, the incidence of this condition is increasing, and it is estimated that by 2040, approximately 300 million people will be affected worldwide. The administration of intravitreal anti-VEGF injections is the gold-standard treatment for the exudative form of age-related macular degeneration. The therapy not only slows down the progression of this chronic disease but also gives patients the chance to achieve a better quality of life and acceptance of the disease, regain visual efficiency from before the onset of the condition, and thus revitalize their psychosocial roles [19,20].

The literature review indicates that there are few studies addressing the topic of acceptance of eye diseases. Most researchers focus on assessing the perception of illness by patients diagnosed with chronic conditions such as hypertension, diabetes, or ulcerative colitis.

Our study showed that almost half of the patients with nAMD had a moderate level of acceptance of the disease, and more than one-third had a low level of acceptance. Receiving a series of seven intravitreal injections was associated with an improvement in overall disease acceptance, as measured by the AIS. The acceptance of the disease in the context of age-related macular degeneration differed before and after intravitreal injections. Before treatment, patients often feel anxiety and uncertainty about the future of their vision, which lowers their level of acceptance of the disease. Fears of disease progression, loss of vision, and their impact on daily life and independence cause stress and depression, making it difficult to cope with the disease. Concerns about the procedure, its effectiveness, and potential side effects may negatively influence acceptance of the disease. However, after starting treatment with intravitreal injections, many patients notice an improvement or stabilization of their vision, which can have a positive impact on their mood and acceptance of the disease. The visible effects of treatment can increase confidence in therapy and reduce the fear of disease progression. Improving vision or preventing deterioration contributes to better quality of life and greater independence, which promotes better acceptance of AMD. However, acceptance of the disease after intravitreal injections may be difficult due to the need for regular visits to an ophthalmologist and continuation of treatment. Patients must receive long-term treatment and regular injections, which can cause stress and discomfort. Nevertheless, awareness of the effectiveness of the therapy and the improvement or stabilization of vision has a positive effect on the acceptance of the disease. When reviewing the available literature on acceptance of the disease among patients with neovascular AMD, no studies addressing this issue have been conducted. Therefore, the results of our study were compared with those of previous studies on other diseases. In the field of ophthalmic diseases, similar results were obtained by authors who measured the acceptance of the disease among patients undergoing cataract surgery by phacoemulsification. More than half of the patients accepted the disease to a medium degree, and almost a quarter to a low degree [21]. Compared with nAMD, senile cataract is a natural degenerative eye disease that can be treated surgically. Disorders that develop in the course of cataracts and impaired vision are reversible in most cases if not accompanied by other ophthalmic diseases. Patient satisfaction after cataract surgery is high and strongly correlated with improved visual function [22,23].

Additionally, Malewicz et al. [24] reported in their studies that the average AIS score for patients diagnosed with cataracts was higher than in our own research. Notably, the authors also observed that patients with a high level of illness acceptance were 2.5 times more likely to adhere to glaucoma therapy. Furthermore, they noted that illness acceptance was an indicator of the degree of adaptation to the disease and was positively correlated with self-esteem, self-efficacy, and engagement in healthy behaviors. This trend emphasizes the importance of psychological factors in disease management and patient outcomes.

The overall disease acceptance rate obtained in our study was similar to that of psoriasis [25,26] and chronic obstructive pulmonary disease [27].

Compared with patients with nAMD, better acceptance of the disease was observed in patients with ulcerative colitis, both in the exacerbation and remission phases of the disease [28], as well as in dialysis patients. In this case, the higher AIS score may have been due to the younger age of the patients, as well as a significant proportion of patients who had undergone prior renal transplantation, which may have significantly reduced the burden of the disease [29].

Higher acceptance of the disease was also noted in patients with Parkinson’s disease [30], osteoarthritis [31], non-malignant lung diseases (asthma, COPD, obstructive sleep apnea) [32], stomach cancer [33], bladder cancer [34], breast cancer, endometrial cancer, ovarian cancer [35], hypertension [36], and type 2 diabetes [37].

A lower acceptance of the disease was observed only in patients diagnosed with diabetic foot syndrome. The authors also showed that the level of disease acceptance significantly correlated with adherence to therapeutic recommendations. Patients with better disease acceptance are less likely to adhere to therapeutic recommendations, which is why it is necessary to constantly monitor patients’ adherence to therapeutic recommendations, regardless of the stage of the disease and the level of its acceptance [38,39].

In a broader clinical context, the correlation between high levels of illness acceptance and better adherence to treatment regimens can be seen across various chronic conditions, such as hypertension, diabetes, and ulcerative colitis. These conditions share common challenges in long-term management, where patient engagement and adherence are crucial for effective outcomes. The positive correlation between illness acceptance and self-esteem, self-efficacy, and engagement in healthy behaviors suggests that interventions aimed at enhancing these psychological attributes could be beneficial in improving patient adherence and health outcomes [22,23,24,25,26,27,40,41,42]. Comparing our findings with the existing literature, we observe a consistent theme: the psychological well-being of patients plays a critical role in their ability to manage chronic illnesses effectively. These behaviors are crucial for managing conditions like nAMD, where timely intervention can prevent further deterioration of vision.

The implications of our findings are particularly relevant in the context of nAMD, where the progressive nature of the disease requires ongoing patient cooperation and adherence to treatment. By fostering a supportive environment that addresses psychological and emotional needs, healthcare providers can enhance patient satisfaction and treatment success. Future research should continue to explore the interplay between psychological factors and disease management, with a focus on developing comprehensive care models that integrate psychological support with medical treatment.

Intravitreal therapy is a relatively new type of treatment. Less than ten years ago, at the end of 2015, a drug program for patients with wet AMD was introduced in Poland. Since then, VEGF inhibitors have become the gold-standard therapy, and studies have shown that treatment with anti-VEGF agents requires continuity and repetition of intravitreal injections, depending on the manifestation of the disease, until the end of the patient’s life [43,44,45].

Both our results and those reported by other authors indicate that patients with neovascular AMD have a poor quality of life and often suffer from anxiety and depressive symptoms. A decrease in quality of life is associated with reduced visual acuity, contrast sensitivity, and metamorphopsia in the course of the disease. Anti-VEGF therapy results in improved visual acuity, contrast sensitivity, and reduced metamorphopsia, which are strong predictors of improved quality of life in patients with nAMD [46].

Intravitreal anti-VEGF injections are an invasive treatment method that carries the risk of complications, including irreversible vision loss. From the patient’s perspective, the diagnosis and treatment of the disease are new and life-changing experiences, and potential complications raise concerns about both starting and continuing therapy. This study provides evidence on the impact of intravitreal therapy on ophthalmic examination outcomes and perceived quality of life. Our findings may help patients decide whether to start treatment for the exudative form of AMD [43,44,45].

Before intravitreal injections, disease acceptance is at a lower level due to fear and uncertainty; however, after starting treatment, it may increase due to the visible benefits of the therapy. Regular treatment and associated challenges may still be a barrier to the full acceptance of AMD. Psychological support and patient education play key roles in the disease acceptance process both before and after treatment initiation. Psychological support such as cognitive–behavioral therapy can help people accept the disease by working through negative thoughts and emotions associated with the diagnosis. Support groups and educational programs that enable patients to understand the disease and share their experiences also accelerate the acceptance process.

The considerations presented in this study have limitations and implications for professional practice. Our study is among the first in Poland to include over 120 patients with AMD, while other studies have recruited significantly fewer patients. The main strength of this study was the use of standardized tools adapted to Polish conditions. Another strength of the study is that the data on ophthalmic diagnostic examination (visual acuity, the presence and severity of metamorphopsia, and contrast sensitivity) were collected by the same person, which translates into the credibility of the study. Another strength of the study is the similar number of representatives of both sexes, which minimized the risk of biased results.

Our study also has some limitations that we hope to avoid in future studies. One limitation is the small sample size, which is not representative of the population of patients diagnosed with age-related macular degeneration. It is worth noting, however, that the studies [21,22,23,26,27,29,33,34,40] to which our study was compared had similar sample sizes. Furthermore, considering that the study was conducted at a single center and that patients were enrolled over a six-month period, the number of respondents was adequate after taking into account the exclusion criteria. Therefore, this limitation did not invalidate the study results.

Another limitation is the method used to collect data on disease acceptance. Although the described research technique is anonymous, it creates the risk of obtaining false answers as a result of conscious or subconscious manipulation of the answers by respondents. This may be due to cognitive errors (response bias and courtesy bias), especially among respondents who required assistance in filling out the questionnaires because of reading problems. This limitation also does not disqualify the results of this study because in the works of other authors [21,24,25,26,27,28,29,30,31,32,33,34,35,36,37,38,40,41], the same research method and technique were used, and individual assistance was provided to the respondents who had difficulties with near vision while filling out the questionnaires.

We suggest that other authors design and conduct further studies in other regions of the country, using the methodology used in our study. This would allow us to obtain more representative data as a basis for a multicenter, cross-sectional study.

## 5. Conclusions

The acceptance of the disease among study participants remained at an average level throughout the course of therapy, from the start of treatment until the completion of a series of seven intravitreal anti-VEGF injections. At baseline, disease acceptance was relatively higher, likely influenced by better corrected visual acuity. After the therapy, acceptance improved further, corresponding to enhanced contrast sensitivity and corrected visual acuity. However, patients presenting visual disturbances on the Amsler grid, both before and after the series of injections, demonstrated lower levels of disease acceptance compared to those without such abnormalities.

This study provides valuable insights into the interplay between visual parameters and disease acceptance in patients with neovascular age-related macular degeneration. These findings may serve as a foundation for larger-scale research investigating the relationships between disease acceptance, the presence and severity of visual disturbances such as metamorphopsia and central scotoma, and other visual parameters before and after intravitreal therapy.

The clinical implications of our study are significant. By enhancing the understanding of the factors influencing disease acceptance, our findings could contribute to the refinement of diagnostic and therapeutic protocols. This, in turn, has the potential to improve patient management and outcomes in individuals diagnosed with AMD.

## Figures and Tables

**Table 1:** General sociodemographic characteristics of the study sample (N = 121).

Sociodemographic Variables		n	%
Sex	female	60	49.59
	male	61	50.41
Age	≤60	1	0.83
	61–65	4	3.31
	66–70	29	23.97
	71–75	29	23.97
	76–80	28	23.14
	81–85	20	16.53
	≥86	10	8.26
Education	20	16.53	6.61
	vocational	48	39.67
	secondary	45	37.19
Marital status	married	77	63.64
	in informal relationship	0	0.00
	single	44	36.36
Place of residence	rural area	7	5.79
	city < 10,000	7	5.79
	city 10,000–100,000	36	29.75
	city > 100,000	71	58.68
Employment status	employed	3	2.48
	employed retiree	13	10.74
	retired	105	86.78

n—number of respondents.

**Table 2:** Characteristics of the study sample with regard to medical variables (N = 121).

Variable		n	%
Eye	left	56	46.28
	right	65	53.72
Smoking	no	103	85.12
	yes	18	14.88
Comorbidities	no	17	14.05
	thyroid diseases	6	4.96
	hypertension	39	32.23
	diabetes	4	3.31
	diabetes + hypertension	32	26.45
	hypertension + other	10	8.26
	other	13	10.74

n—number of respondents.

**Table 3:** Acceptance of the disease according to the AIS.

AIS	M	SD	Me	IQR/2	Min–Max	CV [%]
1	22.12	7.85	21.0	5.50	9–40	35.5
1 (sten point)	1.85	0.74	2.0	0.50	1–3	39.9
2	25.31	7.83	24.0	6.50	11–40	30.9
2 (sten points)	2.08	0.76	2.0	0.50	1–3	36.5

AIS—Acceptance of Illness Scale; M—mean; SD—standard deviation; Me—median; IQR/2—interquartile range; Min—minimum value; Max—maximum value; CV—coefficient of variation; 1—pre-therapy measurement; 2—measurement after seven intravitreal injections.

**Table 4:** The level of acceptance of the disease according to the AIS before and after the therapy.

AIS Levels	AIS				z	*p* *
	1		2			
	n	%	n	%		
Low	43	35.54	30	24.79	4.240	<0.001
Medium	53	43.80	51	42.15		
High	25	20.66	40	33.06		

AIS—Acceptance of Illness Scale; 1—pre-therapy measurement; 2—measurement after seven intravitreal injections; n—number of respondents; z—Wilcoxon test; * *p*—statistical significance according to Wilcoxon test.

**Table 5:** Relationships of visual acuity and contrast sensitivity with acceptance of the disease according to the AIS before the therapy.

Predictor	b	SE	β	Lower 95% CI	Upper 95% CI	t	*p*
Intercept	27.78	4.28	-	-	-	6.490	<0.001
Uncorrected visual acuity	−3.29	2.94	−0.11	−0.30	0.08	−1.120	0.265
Corrected visual acuity	−12.80	4.31	−0.30	−0.51	−0.10	−2.970	0.004
Contrast sensitivity	5.56	2.81	0.17	−4.50 × 10^4^	0.35	1.980	0.051

b—non-standardized regression coefficient; SE—standard error; β—standardized regression coefficient; 95% CI—confidence interval; *p*—statistical significance; t—difference-in-means test with Bonferroni correction for multiple comparisons.

**Table 6:** Relationships of visual acuity and contrast sensitivity with acceptance of the disease according to the AIS after receiving seven intravitreal injections.

Predictor	b	SE	β	Lower 95% CI	Upper 95% CI	t	*p*
Intercept	20.85	4.51	-	-	-	4.626	<0.001
Uncorrected visual acuity	0.10	4.17	0.00	−0.24	0.24	0.023	0.982
Corrected visual acuity	−8.66	3.90	−0.29	−0.54	−0.03	−2.219	0.028
Contrast sensitivity	6.72	2.53	0.27	0.07	0.46	2.661	0.009

b—non-standardized regression coefficient; SE—standard error; β—standardized regression coefficient; 95% CI—confidence interval.

**Table 7:** Relationships of the presence and severity of metamorphopsia and central scotoma (the Amsler test) with acceptance of the disease measured by the AIS before the therapy.

Predictor	b	SE	β	Lower 95% CI	Upper 95% CI	t	*p*
Intercept	26.73	1.96	-	-	-	13.610	<0.001
No metamorphopsia (ref.)	-	-	-	-	-	-	-
Metamorphopsia	−3.69	2.23	−0.47	−1.03	0.09	−1.660	0.100
Central scotoma	−6.98	2.60	−0.89	−1.55	−0.23	−2.690	0.008
Central scotoma and metamorphopsia	−6.67	2.36	−0.85	−1.45	−0.26	−2.830	0.005

b—non-standardized regression coefficient; SE—standard error; β—standardized regression coefficient; 95% CI—confidence interval.

**Table 8:** Relationships of the presence and severity of metamorphopsia and central scotoma (the Amsler test) with acceptance of the disease according to the AIS after seven intravitreal injections.

Predictor	b	SE	β	Lower 95% CI	Upper 95% CI	t	*p*
Intercept	28.04	1.03	-	-	-	27.310	<0.001
No metamorphopsia (ref.)	-	-	-	-	-	-	-
Metamorphopsia	−3.88	1.42	−0.50	−0.85	−0.14	−2.730	0.007
Central scotoma	−8.04	3.47	−1.03	−1.90	−0.15	−2.320	0.022
Central scotoma and metamorphopsia	−9.75	2.98	−1.25	−2.00	−0.49	−3.270	0.001

b—non-standardized regression coefficient; SE—standard error; β—standardized regression coefficient; 95% CI—confidence interval.

## Data Availability

The data presented in this study are available on request from the corresponding author due to privacy reasons.
